# Giant Cell Arteritis With Normal Inflammatory Markers and Progressive Visual Disturbance

**DOI:** 10.7759/cureus.111939

**Published:** 2026-07-02

**Authors:** Saranya Kalyanasundaram Lakshmi, Usman Hassan, Pramodh Hettiarachchi, Deepti Kapur, Kumar Pulupula, Kausik Chatterjee

**Affiliations:** 1 Geriatrics Department, Countess of Chester Hospital NHS Foundation Trust, Chester, GBR; 2 Stroke Department, Countess of Chester Hospital NHS Foundation Trust, Chester, GBR; 3 Rheumatology Department, Countess of Chester Hospital NHS Foundation Trust, Chester, GBR; 4 Radiology Department, Countess of Chester Hospital NHS Foundation Trust, Chester, GBR

**Keywords:** gca, giant cell arteritis, inflammatory markers, multi-modality imaging, normal inflammatory markers, vasculitis, visual disturbance

## Abstract

Giant cell arteritis (GCA) is a large vessel vasculitis classically presenting with headache, jaw claudication, visual disturbance, and raised inflammatory markers. However, atypical presentations occur, and a delay in recognition can lead to irreversible visual loss.

We report a 75-year-old male with sequential ocular involvement in whom erythrocyte sedimentation rate and C-reactive protein remained within normal limits. Carotid/vertebrobasilar imaging showed atherosclerotic change without critical stenosis, and 18F-fluorodeoxyglucose PET demonstrated vertebral arterial uptake consistent with cranial GCA. High-dose corticosteroids were commenced with rheumatology input. Normal inflammatory indices do not exclude GCA. Clinicians should maintain a high index of suspicion when visual symptoms evolve and initiate treatment without delay where clinical probability is high.

## Introduction

Giant cell arteritis (GCA) is a common cause of headaches in patients over 50 years [1]. GCA is the commonest primary vasculitis in older adults and a leading cause of preventable blindness. The diagnosis is straightforward when classical features such as temporal headache, jaw claudication, visual symptoms, systemic features (fever or weight loss), and elevated inflammatory markers and anemia coexist. Yet, atypical stroke and ocular ischemia can be the initial presenting features [1], and the inflammatory markers may be normal in a minority. Delayed diagnosis risks irreversible complications, particularly visual loss. Clinical decision-making should be driven by a high index of suspicion since no single test has perfect sensitivity [2]. Urgent, probability-based treatment is therefore recommended, particularly when vision is threatened [3].

## Case presentation

A 75-year-old gentleman presented to the emergency department with a new left eye central scotoma on a background of recurrent frontal headaches for a duration of three days. After review by the stroke team, he was commenced on dual antiplatelet therapy (DAPT), and carotid imaging was requested, and he was referred to the ophthalmology department. Two days later, the ophthalmology team diagnosed a left central retinal artery occlusion (CRAO). There were no focal neurological deficits on multiple stroke team reviews. Over subsequent weeks, carotid Doppler revealed mild plaques bilaterally without hemodynamically significant stenosis. He was booked for a brain MRI and seven-day ambulatory ECG monitoring and advised not to drive for four weeks.

Six weeks later, he re-presented with blurred vision in the right eye. Ophthalmology examination showed his anterior segments were unremarkable, and examination of the left eye showed evidence of the previously documented CRAO. Dilated fundus examination of the right eye showed attenuated retinal vasculature but no clear signs of an embolus and no evidence of a macular cherry red spot. Ophthalmodynamometry testing was positive for a collapsible central retinal pulse. Intravenous acetazolamide was given by the ophthalmology team. CT angiography (CTA) showed mild-moderate carotid stenoses and a non-dominant right vertebral artery with apparent focal V3-V4 narrowing, reported as likely atheromatous. Antiplatelet therapy was switched from clopidogrel to ticagrelor, and vasculitis work-up was pursued. There were still no limb or speech deficits.

Investigations

Blood tests showed an erythrocyte sedimentation rate (ESR) of 16 mm/h, and autoimmune screen, antiphospholipid antibodies, and immunoglobulins were unremarkable, as shown in Table 1.

**Table 1:** Blood test report. ESR: erythrocyte sedimentation rate; WBC: white blood cells; PLT: platelets; HB: hemoglobin; APCR: activated protein C resistance; MPO: myeloperoxidase; dsDNA: double-stranded DNA.

Parameters	Lab value	Reference range
General hematology		
ESR	16	1 to 20 mm/hr
WBC	7.1	4 to 11 x 10^9/L
PLT	339	120 to 400 x 10^9/L
HB	135	130 to 170 g/L
Neutrophil	5.0	2 to 7.5 x 10^9/L
Anti-phospholipid antibody screen		
APCR	2.78	2.61 to 3.32
Anti-thrombin III activity	110	80 to 125 %
Protein C activity	115	65 to 155 %
Protein S free	64	75 to 145 %
IgG anti-cardiolipin antibodies	20	<=9.9
IgM anti-cardiolipin antibodies	1.7	<=9.9
Autoimmune serology		
Anti-nuclear antibodies	Negative	
PR3	<0.2	<=1.9
Anti-mitochondrial antibodies	Negative	
MPO	<0.2	<=3.4
Anti-gastric parietal cell antibodies	Positive	
dsDNA (screen)	Negative	
Intrinsic factor antibodies	Negative	
Anti-liver-kidney microsomal antibodies	Negative	
Anti-smooth muscle antibodies	Negative	
Immunoglobulin and complement levels		
Immunoglobulin A level, blood	3.96	0.69 to 3.82 g/L
Immunoglobulin G level, blood	10.10	7.2 to 16.9 g/L
Immunoglobulin M level, blood	0.57	0.63 to 2.77 g/L
Complement C3 level, blood	1.48	0.9 to 1.8 g/L
Complement C4 level, blood	0.34	0.1 to 0.4 g/L

CTA of the head and neck demonstrated mild-to-moderate atherosclerotic disease of the internal carotid arteries and focal narrowing of the right V3-V4 vertebral artery segment in the non-dominant vessel (Figures 1, 2). Subsequently, 18F-fluorodeoxyglucose (18F-FDG) positron emission tomography (PET) demonstrated diffuse increased uptake along both vertebral arteries, supporting the diagnosis of cranial GCA (Figure 3). Following PET, the patient had a brain MRI, which showed minor chronic microvascular ischemic change in frontal deep white matter, as shown in Figure 4.

**Figure 1:**
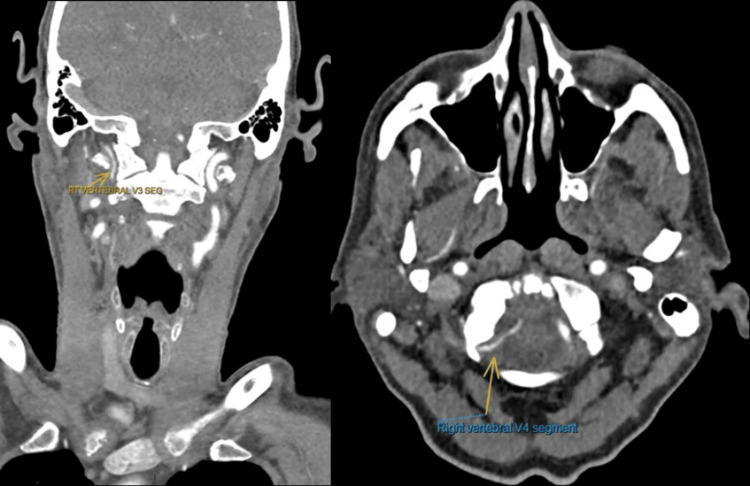
Computed tomography angiography (CTA) of the head/neck showing focal right vertebral V3-V4 narrowing (non-dominant artery).

**Figure 2:**
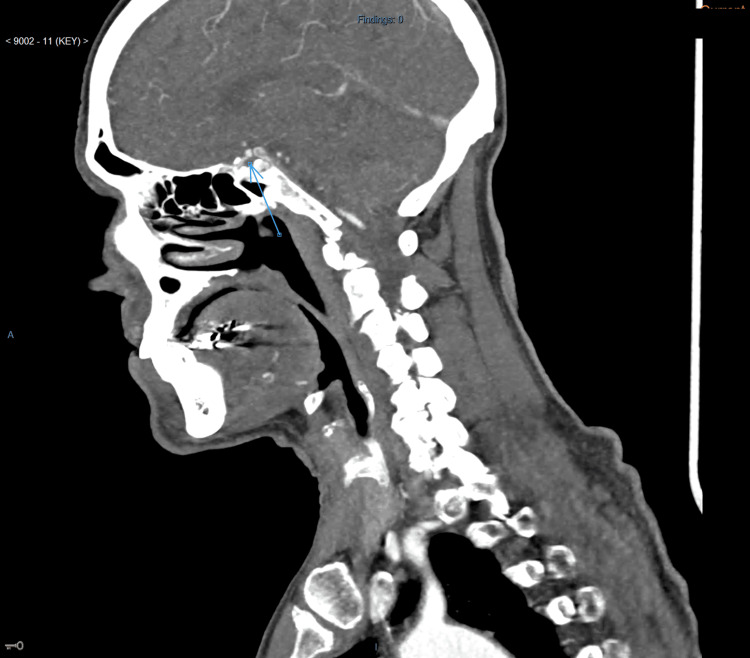
Sagittal CT angiogram showing left supraclinoid internal carotid artery narrowing (arrow). The arrow indicates the left supraclinoid internal carotid artery.

**Figure 3:**
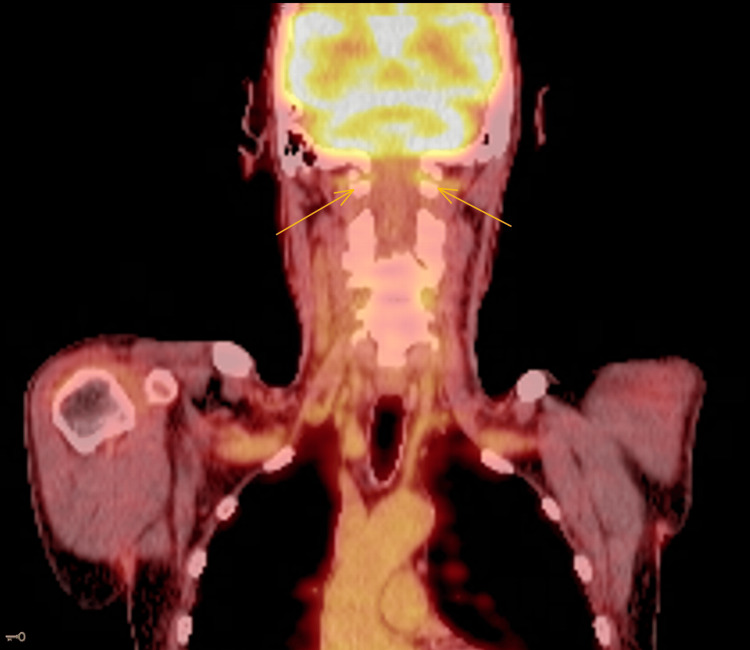
Fused axial image of PET-CT showing increased uptake in both vertebral arteries (arrows).

**Figure 4:**
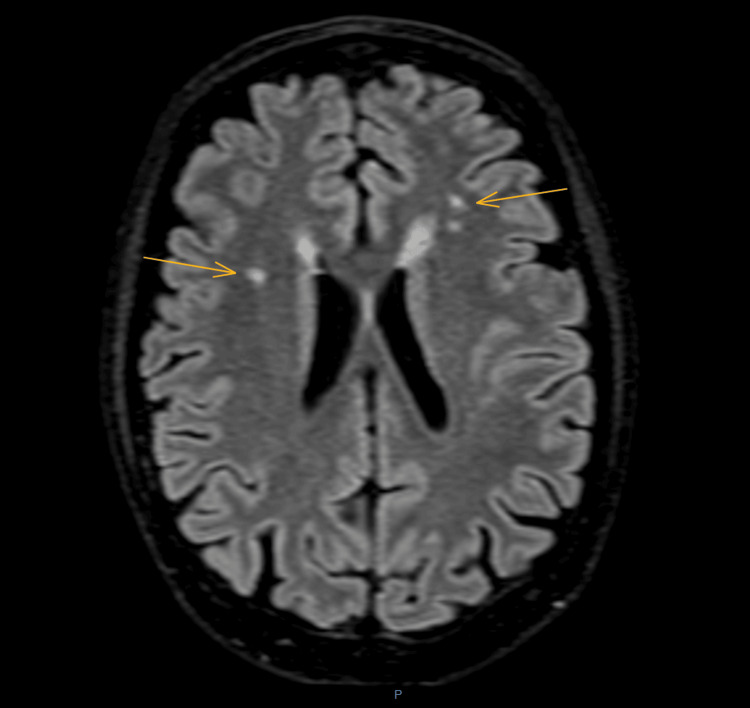
Axial fluid-attenuated inversion recovery (FLAIR) MRI section of the brain showing hyperintense signals in bilateral frontal white matter, suggesting microvascular ischemic changes.

Differential diagnosis

The principal alternative considered was embolic occlusion from atherosclerotic disease of the carotid or aortic arch, given the patient’s age and the initial presentation with CRAO. However, vascular imaging demonstrated only mild-moderate internal carotid atherosclerosis without hemodynamically significant stenosis or unstable plaque to account for sequential ocular events. Cardioembolism was also entertained, but the absence of arrhythmia on initial rhythm monitoring and the lack of structural cardiac findings reduced its likelihood pending longer-term surveillance. Non-arteritic anterior ischemic optic neuropathy (NAION) represents a common cause of acute, painless vision loss in older adults and was considered; nonetheless, the evolving, bilateral pattern of visual disturbance coupled with constitutional cranial symptoms favored an arteritic mechanism. Inflammatory or demyelinating optic neuropathies (including optic neuritis) were felt to be less consistent with the clinical context and age, and there were no systemic features or imaging characteristics to suggest these diagnoses. Hematological causes such as hyperviscosity were screened for but not supported by laboratory testing. Taken together, the chronology of symptoms (CRAO, followed by contralateral visual blurring), the persistence of clinical suspicion despite normal inflammatory markers, and the 18F-FDG PET signal along the vertebral arteries collectively supported a working diagnosis of GCA with normal inflammatory indices, for which urgent treatment was prioritized.

Treatment

Empirical high-dose corticosteroids were initiated urgently (intravenous methylprednisolone 500 mg for three days, followed by oral tapering dose of prednisolone) in consultation with rheumatology, given high clinical probability of GCA and the risk to vision. Antiplatelet therapy was continued, and bone/gastric protection was instituted.

Outcome and follow-up

Visual symptoms stabilized following steroid initiation (initial visual acuity: right eye = 6/4, left eye = 6/5; visual acuity on follow-up after initiation of steroids: right eye = 6/36, left eye = 1/60). Outpatient follow-up with rheumatology and ophthalmology was arranged for steroid tapering, relapse monitoring, and imaging surveillance for large-vessel involvement. The patient was followed up in the rheumatology clinic further, and blood tests were done, which showed normal ESR (2 mm/h), CRP < 1, slightly elevated neutrophils (8.7), and normal white blood cells (9.3). Also, the steroid dose was tapered down to 50 mg for four weeks, then reduced to 40 mg. Further follow-up was arranged with rheumatology in eight weeks. Further outpatient ophthalmology review reported an intraocular pressure (IOP) of 13 mmHg in both eyes, with no neovascularization observed in either eye. As the patient had ocular ischemia as a part of GCA, he was advised regarding the risk of neovascularization and a further rise in IOP. He is kept under the medical retina clinic follow-up.

## Discussion

This case underscores two clinically important atypical features of GCA: (1) normal inflammatory markers and (2) sequential ocular involvement beginning with CRAO. While arteritic anterior ischemic optic neuropathy and visual field defects account for most ocular GCA, CRAO is recognized and may precede further visual compromise. Normal ESR/CRP occurs in a minority, reported in up to 0-22% in systematic reviews [4], so normal indices should not be used to exclude GCA when the pre-test probability is high. Multimodality imaging, particularly 18F-FDG PET, helped support the diagnosis by demonstrating vertebral arterial uptake, highlighting that classification criteria are not diagnostic rules and management must remain probability-based to protect vision.

The 1990 American College of Rheumatology (ACR) criteria [5] comprise the following: age ≥50, new headache, temporal artery abnormality, ESR ≥50 mm/h, and positive temporal artery biopsy; ≥3 factors classify GCA for research. Our patient met two criteria (age, new headache) but had a normal ESR, illustrating that reliance on classification criteria alone could delay vision-saving treatment. This is similar to a study done by Singh et al. [6], where it is stated that elevated inflammatory markers may not always be present in the case of GCA.

In our patient, fundoscopic examination demonstrated features consistent with CRAO, although anterior ischemic optic neuropathy (AION) and visual field loss account for approximately 80-90% of the ocular manifestations of GCA [7]. Our patient reported central scotoma in one eye initially, which later progressed to bilateral visual disturbance. A range of ocular presentations was reported in association with GCA. Kulkarni et al. describe a 73-year-old female who presented to the eye emergency service with diplopia and bilateral choroidal ischemia [7]. Phrathep et al. [8] reported a 60-year-old male presenting to the emergency department (ED) with acute-onset bilateral vision loss and anterior optic neuropathy confirmed on ophthalmic review.

Multiple imaging modalities are available to assess the extent and severity of large-vessel vasculitis (LVV), including ultrasonography, MRI, CT, and 18F-FDG PET. Each modality has advantages and limitations, and the selection is typically guided by the clinical context and local expertise. Current recommendations advise imaging of the aorta and major branches in all patients, including those with predominantly cranial symptoms, as the detection of great vessel involvement may influence treatment strategy and prognosis [9]. In our case, all the above imaging modalities were undertaken as part of our evaluation toward making a diagnosis. MRI of the head showed minor chronic microvascular ischemic change within the deep white matter of both frontal lobes, while PET scan showed diffuse uptake in both vertebral arteries, which is suggestive of cranial GCA in this context, similar to studies done by Anati et al. [10] and Koizumi et al. [11].

Patient’s perspective

On follow-up with our patient, he reported "complete visual loss in one eye and pencil vision in the other, but since starting on steroids, it is static, not worsened."

Learning points

The learning points from this study include not excluding GCA on the basis of normal ESR/CRP when clinical suspicion is high, particularly in the presence of evolving visual symptoms; treating first and confirming later, meaning initiating prompt high-dose steroids when vision is threatened and not awaiting biopsy/imaging if the pre-test probability is high. The study recommends using multimodality imaging (e.g., temporal-artery ultrasound, MRI/CTA, and 18F-FDG PET) to support diagnosis and map large-vessel involvement. CRAO can be an ocular manifestation of GCA and may herald bilateral visual involvement; early recognition can preserve sight.

## Conclusions

GCA can present with atypical symptoms and normal initial investigations. Clinicians should maintain a high index of suspicion in patients with chronic headache and ocular symptoms, given the risk of rapid progression to irreversible vision loss. We also recommend initiating high-dose corticosteroid therapy, with or without antiplatelet therapy, as clinically indicated, as a preventative measure until the final diagnosis is confirmed by further investigations, since treatment delays can result in serious, irreversible visual impairment.
